# Perceptions Toward Telemedicine of Health Care Staff in Nursing Homes in Northern Germany: Cross-Sectional Study

**DOI:** 10.2196/47072

**Published:** 2024-08-07

**Authors:** Pia Traulsen, Lisa Kitschke, Jost Steinhäuser

**Affiliations:** 1 Institute for Family Medicine, University Medical Center Schleswig-Holstein, Campus Lübeck, Luebeck, Germany

**Keywords:** eHealth, telemedicine, nursing home, pandemic, Germany

## Abstract

**Background:**

Digitalization in the German health care system is progressing slowly, even though it offers opportunities for improvement of care. In nursing homes, most of the staff’s work is paper based. Following the pandemic, there has been a decrease in the use of telemedicine applications. To ensure long-term implementation, the views of users, in this case nurses, are of interest.

**Objective:**

This cross-sectional study was conducted to describe which digital applications are already being used at inpatient care facilities, the attitude of nurses toward telemedicine, and for which areas the use of telemedicine in the facilities is considered appropriate by the participants.

**Methods:**

All inpatient care facility staff in Schleswig-Holstein were invited to participate in the survey from August 1 to October 31, 2022. The questionnaire consists of 17 determinants that ask about the attitude, use, and possible applications of telemedicine. In addition to a descriptive analysis, the influence of the general attitude toward telemedicine on various determinants was examined using the Fisher exact test for nominal variables and Spearman correlation coefficient for metric variables.

**Results:**

A total of 425 caregivers participated in the survey. Of these respondents, 10.7% (n=41) currently used video consultations, and 76.1% (n=321) of the respondents were in favor of video consultations being practiced in training. Furthermore, 74.8% (n=312) of the respondents would attend a training on telephone medical consultation. Respondents indicated that video consultations have a small added value compared to asynchronous telemedicine (eg, sending photos). However, video consultations were perceived as somewhat less time-consuming than other communication channels. Video consultations are perceived as most useful for clarifying urgent problems. The respondents estimated that one in five paramedic calls at their facilities could be reduced through telemedicine approaches. It was important to the participants that telemedicine is as simple as possible and that there is a high level of data security.

**Conclusions:**

Although many caregivers have a positive attitude toward telemedicine and perceive its advantages, communication channels such as video consultation are still used infrequently in care facilities. To promote the use of telemedicine applications, it is important to emphasize their benefits. The presumed saving of paramedic calls thus represents a benefit, and it is crucial to train caregivers in the use of telemedicine to avoid uncertainties in dealing with the newer technologies. It is important to give them enough time and repetitions of the training.

## Introduction

The digitalization of the German health care system offers opportunities for the improvement of care. For instance, established communication channels in paper-based form can be eliminated and faster communication between different providers can be strengthened [1,2]. Areas with less access to care can be supported by digital applications [2-4]. The benefits of telemedicine recognized by health care stakeholders include providing better access to care and further communication options [1,5,6]. Telemedicine applications may also help to reduce avoidable ambulance callouts and hospitalizations [7,8].

The term telemedicine is, based on the definition of the German National Association of Statutory Health Insurance Physicians, defined as follows: telemedicine includes applications that can be used for consultation, diagnostics, or therapy over a geographical or temporal distance. This can be done through a wide variety of applications such as video consultation, email, or an app [9].

Nevertheless, digitalization in the German health care system is progressing slowly. Approximately 70% of nursing staff still used paper-based methods. This causes incomplete documentation and is time-consuming [10]. A study from Germany shows that telemedicine applications, such as video consultations, simplify communication between doctors and nurses. Due to flexibilities in time and location, video consultations enable a faster exchange between the doctor and the nursing home [5,11]. Since there is a shortage of nursing staff and a chronic lack of time, the question arises as to why digitalization is not promoted more in this area of care.

In the first year of the COVID-19 pandemic, rapid implementation of telemedicine, and video consultations in particular, was predicted [12]. Nursing homes struggled with isolation rules (eg, shortage of protective clothing). To maintain continuity of care, telemedicine applications were often used during this time [1]. However, it is also apparent that telemedicine applications are being used less after the pandemic, including in nursing homes [13-16].

To successfully implement a telemedicine application in the long-term, it is necessary to explore the attitude of the nursing staff toward telemedicine. In this way, these expectations and perceptions toward telemedicine can be used to tailor the implementation process [13]. Therefore, this cross-sectional study aimed to explore the attitude of caregivers working at inpatient care facilities toward telemedicine, which applications are already used, and what is considered useful.

## Methods

### Study Design and Population

**Figure 1.**
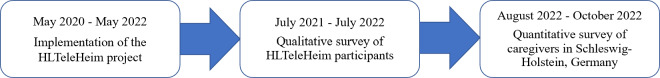
Timeline of the study and evaluation.

In the federal state of Schleswig-Holstein, Germany, the project (HLTeleHeim) was influenced by observations of the challenges in primary care during the COVID-19 pandemic when nursing homes and palliative care providers were performing video consultation with general practitioners, specialists, pastors, or family members. The aim was to support the care of residents during access restrictions in care facilities in the early phase of the SARS-CoV-2 pandemic. The equipment and the training on how to use it was free for the participants [9,13]. Figure 1 provides a better overview of the project’s timeline.

For this cross-sectional study, all 567 inpatient care facilities in Schleswig-Holstein were contacted by mail. The letters contained information about the survey as well as 10 questionnaires and return envelopes. People 18 years or older who were active in providing care could participate in the inquiry. The questionnaires were mailed on August 1, 2022, and could be returned until October 31, 2022. On September 1 and October 1, 2022, reminders were sent to the nursing homes. From September 1, 2022, a web-based version was created on SurveyMonkey as an alternative to the paper-based questionnaire, which should have enabled as many nurses as possible to participate in the survey. Access via a QR code and link to the web-based survey was sent with the first and second reminders. The survey was carried out anonymously; it was not possible to conclude which person or care facility the completed questionnaire originated from.

### Ethical Considerations

The study is a low-risk study since it is not an experimental study. The data were treated in accordance with the General Data Protection Regulation (European Union) 2016/679. Only employees of the study have access to the data contained in the questionnaires, and the data is protected from unauthorized access. Third parties do not have access to the original documents.

The participants were provided with written information before completing the questionnaire and informed that by submitting the questionnaire they were agreeing to participate in the study. They were also notified that it is not possible to subsequently delete the data received, as it is collected anonymously. The participants did not receive any compensation for participating.

The survey of nurses received a positive ethics vote by the ethics committee of the University of Lübeck (22‐095).

### Questionnaire

The questionnaire was developed based on interviews with nurses, physicians, and app developers from a preliminary project and the existing literature [13,17]. It consisted of 17 determinants; the whole questionnaire can be viewed in Multimedia Appendix 1. Those questions aimed to find out which telemedicine devices are used, whether the respondents had received training on the subject during their education, what the general attitude toward telemedicine is, and for which areas telemedicine devices would be suitable to use. It was also intended to find out which factors (eg, data protection aspects or costs) are particularly relevant in relation to telemedicine. Respondents were also allowed to write their comments on the topic in a free-text section. The questionnaire was pilot-tested for comprehensibility. Therefore, carers from the researchers’ private circle of acquaintances were asked to complete the questionnaire and explain in a discussion round how the questions were perceived and what changes they would suggest. The paper-based questionnaire contains the same questions as the web-based questionnaire, so all responses were analyzed together.

### Analyses

Data analysis was conducted using the IBM SPSS Statistics 28 program. A descriptive analysis of the data was performed. In addition, the influence of the general attitude toward telemedicine on various determinants was examined. For this purpose, the variable attitude toward telemedicine was summarized as a dichotomous variable. Values 1‐3 (mostly positive to very positive) were defined as a positive attitude and values 4‐6 (mostly negative to very negative) as a negative attitude. The prerequisites for a chi-square test were not fulfilled; therefore, the Fisher exact test was used for nominal variables. For metric variables, Spearman correlation coefficient was calculated. The original variable of the attitude toward telemedicine with 6 expressions was used for this purpose; furthermore, the age variable was created based on the year of birth variable. The significance level was set at .05.

## Results

A total of 425 caregivers participated in the survey—22 of them completed the web-based survey. Table 1 shows the sociodemographic data of the sample.

In the open question for which scenarios the use of video consultation would also be useful, the following points were indicated: ordering medications and bandages, electrocardiograms, specialist consultations, discussions between physicians and residents, communication with guardians, new admissions, avoidance of hospitalizations, contact with training institutes, and clarification of queries. Three participants responded that video consultations would not be useful in general.

Table 2 shows the current use of telemedicine in nursing homes and for which areas the use of video consultation would be useful.

Table 3 illustrates the general attitude toward telemedicine, how the participants deal with the video consultation, and which aspects are particularly relevant to them.

**Table 1.** Sociodemographic data of the participating nursing staff from Schleswig-Holstein (N=425).

Variable		Participants
Age (years), mean (SD)		43.7 (12.2)
**Sex, n (%)**		
	Female	309 (72.8)
	Male	105 (24.6)
	Missing	11 (2.6)
**Active in care, n (%)**		
	Yes	330 (77.6)
	No	84 (19.8)
	Missing	11 (2.6)
**Certified nurse (n=322), n (%)**		
	Yes	268 (63.1)
	No	54 (12.6)
	Not active in care	90 (21.2)
	Missing	13 (3.1)
Work experience (years), mean (SD)		16.1 (10.6)
**Location of the nursing facility, n (%)**		
	Rural	226 (53.2)
	Urban	186 (43.7)
	Missing	13 (3.1)

**Table 2.** Current use and benefits of telemedicine of the participating nursing staff from Schleswig-Holstein (N=425).

Question		Yes, n (%)	No, n (%)	Missing, n (%)
**During the last 12 months, the possibilities for using telemedicine have been...**				
	...addressed within the team?	112 (26.4)	309 (72.7)	4 (0.9)
	...addressed by the residents?	36 (8.5)	383 (90.1)	6 (1.4)
	...addressed by external contacts?	162 (38.1)	257 (60.5)	6 (1.4)
Have you been trained in videoconferencing during vocational training?		76 (17.9)	349 (82.1)	0 (0.0)
Do you think that videoconferencing should be practiced during vocational training?		321 (75.5)	101 (23.8)	3 (0.7)
Would you attend an advanced training course on medical telephone consultation?		312 (73.4)	105 (24.7)	8 (1.9)
Do you document vital signs in a computer system at your facility?		329 (77.4)	93 (21.9)	3 (0.7)
**Use in work**				
	Fax	392 (92.2)	22 (5.2)	11 (2.6)
	Telephone	406 (95.5)	13 (3.1)	6 (1.4)
	Messaging app	57 (13.4)	310 (72.9)	58 (13.7)
	Emails	300 (70.6)	104 (24.5)	21 (4.9)
	Video consultation	41 (9.6)	341 (80.2)	43 (10.2)
**Do you find the use of video consultations useful to/for...**				
	...clarify in urgent issues?	300 (70.6)	117 (27.5)	8 (1.9)
	...discuss new findings that have arisen?	244 (57.4)	173 (40.7)	8 (1.9)
	...progress checks?	238 (56.0)	179 (42.1)	8 (1.9)
	...discuss lab results?	219 (51.5)	198 (46.6)	8 (1.9)
	...conversations between residents and family members?	190 (44.7)	226 (53.2)	9 (2.1)
	...routine house calls?	158 (37.2)	259 (60.9)	8 (1.9)

**Table 3.** Attitude and relevant aspects of telemedicine of the participating nursing staff from Schleswig-Holstein (N=425).

Question		Score^a^		Missing, n (%)
		Median	IQR	
How is your general attitude toward telemedicine?		2	2-3	11 (2.6)
**If you use video consultations**				
	How confident do you feel in using them?	2	2-3	8 (1.8)
	In your experience, how big is the additional benefit a video consultation offers compared to sending an image for health issues relating to a resident? (*n*=37)	3	2-4	6 (1.4)
	How would you rate the time aspect of video consultations compared to previous forms of communication? (*n*=37)	3	1-3.5	6 (1.4)
**How relevant is/are...**				
	...personal contact with doctors to clarify a question for your work?	1	1-2	2 (0.5)
	...personal contact with doctors for the residents?	1	1-2	2 (0.5)
	...digital communication for your work?	2	1-3	4 (0.9)
	...the cost-to-revenue aspect for you?	3	2-4	20 (4.7)
	...easy usability of a telemedicine workstation for you?	2	1-3	7 (1.6)
	...low costs for hardware and software for you?	3	1-4	20 (4.7)
	...clearly structured organizational processes for you?	2	1-2	9 (2.1)
	...data security aspects for you?	1	1-2	7 (1.6)
	...software compatibility with the information system for you?	2	1-3	16 (3.8)
	...it for you to have a contact person for IT issues?	1	1-2	10 (2.4)
How big was the influence of the COVID-19 pandemic on your day-to-day work in terms of the use of digital media?		2	1-4	8 (1.9)
How big is the influence of the staffing ratio for you when using videoconferencing systems?		3	2-5	21 (4.9)

a 1=very high/positive/relevant; 6=very low/negative/irrelevant.

When asked how much time the participants could spend per shift familiarizing themselves with topics such as telemedicine, an average of 28 (SD 26, median 30, min-max 0-180) minutes was determined. When estimating how long it would take to familiarize themselves with a telemedicine workplace, an average of 47 (SD 76, median 30, min-max 0-1000) minutes was reported.

On average, the emergency service was alerted 5 (SD 9, median 3, min-max 0-135) times per month per area of operation in the nursing homes. Among these, the nurses estimate that on average 1 (SD 2, median 0, min-max 0-15) of these emergency operations could be spared by telemedicine.

Looking at the Fisher exact test, there are differences (*P*<.001) between the positive and negative responses to the questions “Do you think video consultation should be practiced in training?” and “Would you attend training on medical telephone consultation?” There were differences in the questions about whether video consultation is considered useful for conversations between residents and relatives (*P*=.002) and routine examinations (*P*=.03). Those with a negative attitude were more likely to answer “no” to these questions. There were no differences between the groups in the questions about whether video consultation is considered useful for discussing laboratory results and for new findings. There was a difference (*P*<.001) in the question about whether video consultation is considered useful for follow-up checks. Those with a positive attitude were more likely to answer “yes” to these questions, while those with a negative attitude were more likely to answer “no.” Furthermore, there was a difference (*P*=.003) in the question about whether video consultation would be useful to clarify urgent questions. Those with a positive attitude were more likely to answer “yes” to this question. There were no differences in attitudes toward telemedicine based on the location of the nursing facility. In both rural and urban facilities, about 80% had a positive attitude toward telemedicine.

The calculation with Spearman ρ showed no correlation (0.079) between age and attitude toward telemedicine (*P*=.11). There was a significant (*P*<.001) negative correlation (−0.274) between attitude toward telemedicine and opinion of how many emergency service calls can be reduced per month.

## Discussion

### Principal Findings

This cross-sectional study aimed to describe which telemedicine devices are already being used at inpatient care facilities and to determine the attitude of nurses toward telemedicine and for which areas the use of telemedicine in the facilities is considered sensible by the nurses.

The survey shows that the telephone and the fax machine are the most frequently used digital tools among nurses in Schleswig-Holstein. Video consultations and messaging apps, on the other hand, are currently used less. According to the PraxisBarometer 2021, video consultations are used by 20% of contract physicians (in Germany there is no obligation to use telemedicine [18]). This may influence the use of video consultations in care facilities [19]. The higher use of the telephone in contrast to video consultation could be explained by the fact that telephone calls are currently faster to execute and that many questions can be answered through a telephone call [20]. This was not asked in the study and could be the subject of further research.

Few participants were educated in using video consultation in their training, but many consider training to be useful. This indicates that training should focus more on digital topics, such as how to conduct video consultations. This is in line with observations from other countries. In the United Kingdom, there is also a need for more training in online communication skills [21]. Further work is needed to determine whether training should and can be adapted in Germany. The large variance in the responses as to how much time is presumably required for familiarization indicates that the training and familiarization times must be individually adapted to the needs of the nurses.

Notably, video consultations are considered more useful for urgent cases and questions than for regular visits. Participants also noted that telemedicine applications could save around 1 emergency ambulance call per month. This is also consistent with observations from other studies [7,8]. In past research, video consultations were considered useful, especially for frequently needed follow-up appointments [5], for instance in the treatment of depression. However, this study was conducted with doctors and not with nurses. There seem to be different views on the appropriateness of video consultations [6,22]. Furthermore, it is noticeable in the survey that for the use of video consultations, more organizational/administrative topics are considered useful, such as ordering medication or communication with training facilities, than direct patient care. It was not possible to determine why nurses prefer video consultation for administrative activities, which could be further researched.

The general attitude of the participants was positive toward telemedicine. Nevertheless, personal contact with physicians was relevant for the respondents. Particular care should always be taken to ensure that video consultations are only used where they really can support care and not completely replace personal contact [11]. The age of the respondents and the location of the care facilities did not influence their attitude toward telemedicine. Therefore, younger people are not the only age group interested in using telemedicine [19,23].

According to the respondents, the COVID-19 pandemic had a strong influence on their use of digital media. This is consistent with other literature. The increased use may be attributed to the fact that in some cases digital solutions were better than no contact at all [24-28]. Whether this influence was perceived positively or negatively cannot be determined. A decrease in video consultations after the relaxation of restrictions during the pandemic has already been seen in physicians’ practices [15,19]. Additionally, whether the influence will be long-lasting could not be determined.

Data protection and a contact person in case of technical problems are very important for the respondents. The compatibility of the systems is also a relevant point for the caregivers. Furthermore, a telemedicine workstation should be as easy to use as possible. The cost aspect is somewhat less relevant for the caregivers themselves. This would presumably be an aspect that is more relevant at the institution level, but this was not addressed in this study. In a possible implementation of telemedicine applications in full inpatient care facilities, these points should be taken into consideration to achieve more success [7,11]. Likewise, efforts should be made to ensure that nurses have enough time to familiarize themselves with telemedicine workstations [8,16]. The respondents in this study suspected that familiarization would take more time than they had available. General attitudes toward telemedicine should also be considered, as the study showed that respondents who were positive about telemedicine were more likely to consider attending further training on the subject, among other things. It is important to keep demonstrating the benefits of telemedicine applications to maximize the motivation to use them [27,29]. Uncertainties about new technologies should be addressed so that people feel comfortable and confident using them [30]. Additionally, the implementations should be designed for the relevant facilities so that they can be used as easily as possible, and familiarization provides more benefits [31]. New applications cannot be implemented if there is no acceptance from the nursing staff [29,32,33].

### Strength and Limitations

A strength of this study is that all 567 full inpatient care facilities in Schleswig-Holstein were contacted. It is not possible to conclude how many institutions participated, as the questionnaires were sent anonymously, and the response rate could not be determined. Since significantly more paper-based questionnaires were completed than web-based questionnaires, it is possible that more people could have been reached if more questionnaires had been sent out per facility. Despite the anonymity of the questionnaires, it is possible that the participants were biased toward answers they perceived to be desirable. In addition, recall bias, especially for the question about emergency services, was possible and must be considered in the interpretation of our data. It is also important to consider that people who are interested in the topic of telemedicine were more inclined to participate in the survey [34,35].

### Conclusion

Attitudes toward telemedicine among nurses in full inpatient care facilities were generally positive. However, newer telemedicine applications, such as video consultation or messaging apps, are infrequently used. The need for telemedicine is seen mainly for acute issues. For the future practice of telemedicine, related content and training should be included in the education of future caregivers. When implementing telemedicine, the specific needs of the users must be considered to be successful. For example, regular customized staff training could be helpful in this regard. Furthermore, caregivers need to be given time for this training. Finally, caregivers need to be involved in the training content.

## Supplementary material

10.2196/47072 Multimedia Appendix 1 Questionnaire.
