# Supplementary material for: Perceptions Toward Telemedicine of Health Care Staff in Nursing Homes in Northern Germany: Cross-Sectional Study
Source: JMIR Aging. 2024 Aug 7;7:e47072. doi: 10.2196/47072 (PMC11322793; doi:10.2196/47072)
Supplement: Multimedia Appendix 1 [file aging-v7-e47072-s001.docx]

**Definition telemedicine:**

In the following, telemedicine refers to devices that can be used for consultation, diagnostics or therapy over a distance in time or space. This can be done using a wide variety of devices such as video consultation, e-mail or via an app.^1^

|  | Very positive Very negative | | | | | |
| --- | --- | --- | --- | --- | --- | --- |
| **1.** What is your general attitude towards telemedicine? | ❑ | ❑ | ❑ | ❑ | ❑ | ❑ |

| **2.** During the last 12 months, the possibilities for using telemedicine have been... | Yes | No |
| --- | --- | --- |
| a) ... addressed within the team? | ❑ | ❑ |
| b) ... addressed by the residents? | ❑ | ❑ |
| c) ... addressed by external contacts (e.g., medical practices, wound managers, palliative care, etc.)? | ❑ | ❑ |
| **3.** Have you been trained in video conferencing during your vocational training? | ❑ | ❑ |
| **4.** Do you think that video conferencing should be practiced during vocational training? | ❑ | ❑ |
| **5.** Would you attend a training course on medical telephone consultations? | ❑ | ❑ |
| **6.** Do you document vital signs in a computer system at your facility? | ❑ | ❑ |
| **7.** Which telemedicine devices do you use at work? |  | |
| a) Fax | ❑ | ❑ |
| b) Telephone | ❑ | ❑ |
| c) Messaging-App (e.g., WhatsApp, Siilo) | ❑ | ❑ |
| d) E-Mail | ❑ | ❑ |
| e) Video conferencing (e.g., Arztkonsultation, Patientus) | ❑ | ❑ |

| **8.** If you use video consultations...:  (otherwise continue with question 9) | Very confident Very insecure | | | | | | | | |
| --- | --- | --- | --- | --- | --- | --- | --- | --- | --- |
| a) ... how confident do you feel in using it? | ❑ | ❑ | ❑ | | | ❑ | | ❑ | ❑ |
|  | Very big Very small | | | | | | | | |
| b) … in your experience, how big is the additional benefit a video consultation offers compared to sending an image for health issues relating to a resident? | ❑ | ❑ | ❑ | | | ❑ | | ❑ | ❑ |
|  | It saves time | | |  |  | | It doesn’t save time | | |
| c)...how would you rate the time aspect of video consultations compared to previous forms of communication (e.g., telephone, home visit)? | ❑ | ❑ | ❑ | | | ❑ | | ❑ | ❑ |

| **9.** How relevant is/are… | Very relevant Irrelevant | | | | | |
| --- | --- | --- | --- | --- | --- | --- |
| a) ...a personal contact with doctors to clarify a question for your work? | ❑ | ❑ | ❑ | ❑ | ❑ | ❑ |
| b) ... personal contact with doctors for the residents? | ❑ | ❑ | ❑ | ❑ | ❑ | ❑ |
| c) ... digital communication for your work? | ❑ | ❑ | ❑ | ❑ | ❑ | ❑ |
| d) ... the cost-to-revenue aspect for you? | ❑ | ❑ | ❑ | ❑ | ❑ | ❑ |
| e) ... easy usability of a telemedical workstation for you? | ❑ | ❑ | ❑ | ❑ | ❑ | ❑ |
| f) ... low costs for hardware and software? | ❑ | ❑ | ❑ | ❑ | ❑ | ❑ |
| g) ... clearly structured organizational processes (e.g., timing of councils)? | ❑ | ❑ | ❑ | ❑ | ❑ | ❑ |
| h) ...the aspects of data security for you? | ❑ | ❑ | ❑ | ❑ | ❑ | ❑ |
| i) ... software compatibility with the information system? | ❑ | ❑ | ❑ | ❑ | ❑ | ❑ |
| j) ... a direct contact for IT questions? | ❑ | ❑ | ❑ | ❑ | ❑ | ❑ |
|  |  |  |  |  |  |  |

|  | Big influence No influence | | | | | |
| --- | --- | --- | --- | --- | --- | --- |
| **10.** How big was the influence of the coronavirus pandemic on your day-to-day work in terms of the use of digital media? | ❑ | ❑ | ❑ | ❑ | ❑ | ❑ |
| **11.** How big is the influence of the staffing ratio for you when using video conferencing systems? | ❑ | ❑ | ❑ | ❑ | ❑ | ❑ |

| **12.** For which scenarios do you find the use of video consultations useful?  (multiple answers possible) | |
| --- | --- |
| a) Clarify urgent issues | ❑ |
| b) Discuss new findings | ❑ |
| c) Routine house calls (visits) | ❑ |
| d) Follow-ups (e.g., wounds) | ❑ |
| e) Discussing laboratory results | ❑ |
| f) Conversations between residents and relatives | ❑ |
| g) Others (please note): | |

|  | In minutes |
| --- | --- |
| **13.** How much time are you able to spend per shift to further educate yourself on topics such as telemedicine? |  |
| **14.** How do you estimate your learning effort to be able to use a telemedical workplace (e.g., starting a video call)? |  |

|  | Quantity per month |
| --- | --- |
| **15.** On average how often do you have to alert the emergency services in your area of operation (e.g., residential area) per month? |  |
| **16.** In your opinion, how many of these emergency service operations could be prevented through the use of telemedicine applications (e.g., video consultations)? |  |

**17.** Personal data:

a) What gender were you assigned at birth? ❑ male ❑ female

b) Which year were you born?

c) Do you work directly in care? ❑ yes ❑ no (continue with question e)

d) If yes: Are you a registered nurse (e.g., 3-year training)? ❑ yes ❑ no

e) How many years have you been working in a nursing home?

f) Your care facility is located... ❑ ...rather rural ❑ ...rather urban

| **Remarks: (please write clearly)** |
| --- |
|  |
